# Antipyrine in Ophthalmia

**Published:** 1898-09

**Authors:** 


Antipyrine in Ophthalmia. In those cases of ophthalmia in which the discharge is profuse and the pain and inflammation are great, a 25 per cent. solution of antipyrine, used three times daily, is said to afford great relief. This is due in a great measure, no doubt, to the antiseptic and analgesic virtues of the drug.

